# Phenotypic analysis of α1,2-mannosidase-like protein deletion mutants in *Saccharomyces cerevisiae*

**DOI:** 10.17912/micropub.biology.000640

**Published:** 2022-09-23

**Authors:** Seita Nakamasu, Takashi Kikuma, Yuji Hashiguchi, Sato Tada, Kanae Sano, Yukishige Ito, Yoichi Takeda

**Affiliations:** 1 College of Life Sciences, Ritsumeikan University; 2 Graduate School of Science, Osaka University; 3 RIKEN Cluster for Pioneering Research

## Abstract

α1,2‑mannosidase-like proteins mediate quality control of glycoproteins in the endoplasmic reticulum. This study explored α1,2‑mannosidase-like protein functions in *Saccharomyces cerevisiae.* Single disruptants in targeted protein-coding genes were found to be viable; however, deletion of *MNL2* resulted in declined yeast growth at 37 °C. The normal growth rate was recovered in double-deletion strains where one of the deletions was in *MNS1* . We also measured the mannosidase activity of microsomal fractions of deficient strains using artificial glycan. Increased mannose trimming activities were demonstrated by the microsomes of *MNL2* -deletion strains compared to levels of activity exhibited by the microsomes of the control strain.

**Figure 1. Phenotypic analysis of α1,2-mannosidase-like protein deletion mutants.**
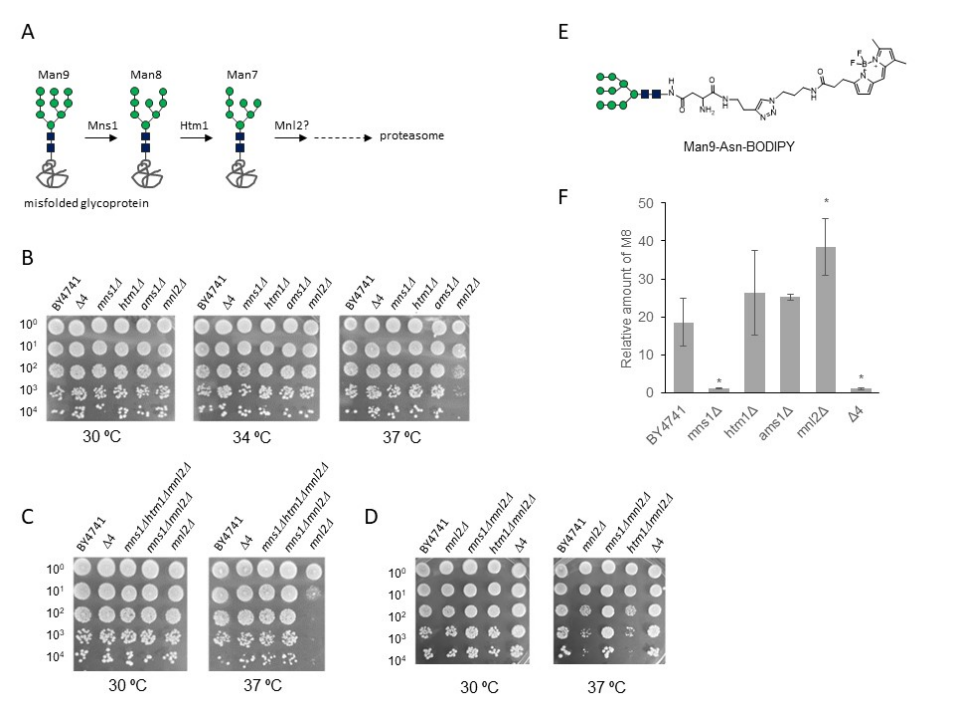
(A) Schematic illustration of mannose trimming by α1,2‑mannosidase-like protein in the ER. (B) Temperature sensitivity of single and quadruple (Δ4) disruptants in the four α1,2 mannosidase-like protein genes. (C) Temperature sensitivity of *mns1* Δ *mnl2* Δ and *mns1* Δ *htm1* Δ *mnl2* Δ compared to that of the single ( *mnl2* Δ) and quadruple (Δ4) disruptants. (D) Temperature sensitivity of *htm1* Δ *mnl2* Δ compared to that of single ( *mnl2* Δ), double ( *mns1* Δ *mnl2* Δ), and quadruple (Δ4) disruptants. (E) Structures of Man9-Asn-BODIPY. (F) Mannose trimming of glycan substrate by microsomal fractions of deficient strains. Bars, mean values. Two-tailed t-test. *p<0.05.

## Description

The endoplasmic reticulum (ER) is the site of the synthesis and folding of membrane and secretory proteins. Misfolded proteins are retro-translocated from the ER to the cytoplasm, where they are degraded by the ubiquitin-proteasome system by a mechanism called ER-associated degradation (ERAD) (Brodsky 2012). The ERAD pathway can be either glycan-dependent or ‑independent (Ninagawa et al. 2021). In the ERAD of glycoproteins in *Saccharomyces cerevisiae* , α1,2‑mannosidase-like proteins cleave mannose residues from misfolded glycoproteins before they are translocated to the cytosol for proteasomal degradation (Quan et al. 2008). Mns1, Htm1, Mnl2, Ams1 are α1,2‑mannosidase-like proteins that are found in *S. cerevisiae* . Mns1 and Htm1 cleave Man9 (Jakob et al. 1998) and Man8 (Pfeiffer et al. 2016), respectively (Figure 1A). Mnl2 is believed to be a mannosidase because ERAD efficiency is reduced in Mnl2-deficient *S. cerevisiae* strains (Martinez Benitez et al. 2011). However, the mannosidase activity of Mnl2 has not been observed *in vitro* . Ams1 is a mannosidase localized to the vacuoles (Yoshihisa and Anraku 1989).

We found that single disruptants in the four α1,2‑mannosidase-like protein genes were viable in the YPD medium at 30 °C, 34 °C, and 37 °C, as was the intact BY4741 strain. The *mnl2* Δ strain exhibited a slow rate of growth at 37 °C (Figure 1B). However, the quadruple disruptant (Δ4) did not demonstrate a decline in growth rate. To identify genes that could override the temperature sensitivity at 37 °C caused by the deletion of *MNL2* , multiple disruptants including *mnl2* Δ were tested for sensitivity to different temperatures. Interestingly, among all the double and triple disruptants, only *mns1* Δ *mnl2* Δ was resistant to the elevated temperature (Figure 1C, D). These results indicated that Mns1 inactivation relieved the growth-restricting effects of disruption of *MNL2* . Mns1 is thought to function upstream of glycan-dependent ERAD; whereas Mnl2 functions downstream of this pathway. When Mns1 is inactivated, the Htm1-dependent pathway may be functional, wherein Htm1 recognizes the Man9 glycoform and trims mannose residues to enable their recognition by Yos9, the lectin that regulates ERAD. (Hosomi et al. 2010). Alternatively, involvement of unconventional protein secretion (UPS) pathway would be possible, as, in mammalian cells, TMED complex that mediates UPS of several glycoproteins was reported to recognize the Man9 glycoform as the prime substrate (Park et al. 2022). The results indicate that this alternative pathway may function via a mechanism similar to that of the UPS pathway, especially when both *MNS1* and *HTM1* are deleted.

Subsequently, we analyzed the mannose trimming activities of the microsomal fractions extracted from the disruptants using the fluorescently labeled glycan substrate (Man9-Asn-BODIPY, Figure 1E). The mannose trimming reaction proceeded linearly up to 72 hours although the reaction rate was low. As anticipated, microsomal fractions of the BY4741 strain and disruptants, except *mns1* Δ and Δ4, showed mannose trimming activity. However no further trimming beyond Man8 was observed in any case, and microsomal fractions of the *htm1* Δ did not exhibit specific changes in mannose trimming activity (Figure 1F). These results would indicate that the non-proteinic substrate might not be suitable to evaluate the activity of Htm1. Additionally, no significant effect of the Ams1 disruption on mannose trimming was observed, presumably because Ams1 is a vacuole-localized protein, and not able to affect the mannose trimming activity in the ER. On the other hand, magnitudes of the mannose trimming activity exhibited by the *mnl2* Δ strain was markedly higher than other strains. These results would be interesting and warrant further investigation to address the possibility of mutual intervention of ER mannosidases in *S. cerevisiae* .

## Methods

*Construction of disruptants*

Single disruptants were purchased from Horizon Discovery Biosciences Limited (Cambridge, Great Britain). Other disruptants were generated by the replacement of a target gene with a marker gene via homologous recombination. Plasmids harboring α1,2‑mannosidase gene deletion cassettes were constructed by inserting a marker gene, and 500 bp upstream and downstream of target genes into pDEST™R4-R3 (Thermo Fisher Scientific, Waltham, MA) using the MultiSite Gateway cloning system (Thermo Fisher Scientific). The deletion cassettes were amplified by polymerase chain reaction (PCR) using these plasmids as templates and then were used for the transformation with the Quick & Easy Yeast Transformation Mix (Takara Bio, Kusatsu, Japan).

The double disruptant *mns1* Δ *mnl2* Δ and *htm1* Δ *mnl2* Δ were constructed by the replacement of *MNS1* and *HTM* , respectively, with an auxotrophic marker, *URA3,* in the *mnl2* Δ strain. The Δ3 strain was generated from the *htm1* Δ strain; this was achieved through the replacement of *MNS1* and *MNL2* with *URA3* and *LEU2* , respectively. The Δ4 strain was constructed by replacement of *AMS1* with an auxotrophic marker, *MET15,* in the Δ3 strain

*Cell culture*

Yeast cells were first cultured on YPD agar medium at 30 °C for 2–3 days. The cells were then subcultured in 5 mL YPD liquid medium and incubated overnight. This liquid culture was subsequently added to 200 mL of YPD liquid medium and incubated at 30 °C using an orbital shaker incubator. The yeast cells were allowed to grow until the culture reached an OD _600_ value of 0.6. The cells were then collected by centrifugation (8,000 × *g* , 4 °C, 10 min). The recovered cells were frozen in liquid nitrogen, stored at -80 °C, and thawed on ice before use.

*Preparation of microsomal solution*

To prepare the microsomal solution, we added 5 mL of Yeast Buster reagent (Merck, Germany) and 50 µL of 100X THP (Merck, Germany) solution to 1 g of the recovered cells. This solution was stirred slowly at room temperature for 30 min and then centrifuged (16,000 × *g* , 4 °C, 10 min). The collected supernatant was ultracentrifuged (100,000 × *g* , 4 °C, 60 min). The resultant pellet was dissolved in 100 µL of MES lysis buffer (20 mM MES, 150 mM NaCl, 5 mM CaCl _2_ , and 0.05% Triton X-100; pH = 5.5). Before performing the mannose trimming assays, the microsomal solutions were diluted using 20 mM MES lysis buffer to equalize their total protein contents. The total protein concentrations of the microsomal solutions were determined using the Bio-Rad Protein Assay Dye Reagent Concentrate (Bio-Rad, CA).

*Mannose trimming assay*

The synthetic glycan substrate Man9-Asn-BODIPY (Figure 1E) was added to 20 µL of the microsomal solution and the mixture was incubated at 30 °C for 72 h. The reaction was stopped by adding acetonitrile (twice the volume of the reaction solution). The enzymatic reactions were analyzed using the method described in (Kikuma et al. 2022).

## Reagents

**Table:** 

**Medium**	**Composition (per liter)**
YPD	Yeast Extract 10 g, poly-peptone 20 g, glucose 20 g
SD-Ura	Minimal SD Base (Takara) 26.7 g, -His/-Trp/-Leu/-Ura Do Supplement (Takara) 0.60 g, L-Histidine 76 mg, L-Tryptophan 76 mg, L-Leucine 380 mg
SD-His	Minimal SD Base (Takara) 26.7 g, -His/-Trp/-Leu/-Ura Do Supplement (Takara) 0.60 g, L-Tryptophan 76 mg, L-Leucine 380 mg, L-Uracil 76 mg

**Table:** 

Strain	**Genotype**	Source
BY4741	*MAT* **a** *his3* Δ *1 leu2* Δ *0 met15* Δ *0 ura3* Δ *0*	Horizon Discovery Biosciences Limited
*mns1* Δ	*MAT* **a** *his3* Δ *1 leu2* Δ *0 met15* Δ *0 ura3* Δ *0* *mns1* :: *KanMX*	Horizon Discovery Biosciences Limited
*htm1* Δ	*MAT* **a** *his3* Δ *1 leu2* Δ *0 met15* Δ *0 ura3* Δ *0 htm1* Δ:: *KanMX*	Horizon Discovery Biosciences Limited
*mnl2* Δ	*MAT* **a** *his3* Δ *1 leu2* Δ *0* *met15* Δ *0 ura3* Δ *0 mnl2* Δ:: *KanMX*	Horizon Discovery Biosciences Limited
*ams1* Δ	*MAT* **a** *his3* Δ *1 leu2* Δ *0 met15* Δ *0* *ura3* Δ *0 ams1­* Δ::­ *KanMX*	Horizon Discovery Biosciences Limited
*mns1* Δ *mnl2* Δ	*MAT* **a** *his3* Δ *1 leu2* Δ *0* *met15* Δ *0* *mns1* Δ:: *URA3 mnl2* Δ:: *KanMX*	This study
*htm1* Δ *mnl2* Δ	*MAT* **a** *leu2* Δ0 *met15* Δ0 *htm1* Δ:: *HIS3 mnl2* Δ:: *KanMX*	This study
Δ3( *mns1* Δ *htm1* Δ *mnl2* Δ)	*MAT* **a** *his3* Δ *1 mns1* Δ:: *URA3 htm1Δ* :: *KanMX mnl2* Δ:: *LEU2 met15* Δ0	This study
Δ4( *mns1* Δ *htm1* Δ *mnl2* Δ *ams1* Δ)	*MAT* **a** *his3* Δ *1 mns1* Δ:: *URA3 htm1Δ* :: *KanMX mnl2* Δ:: *LEU2 ams1* Δ­::­­­ *MET15*	This study
